# Local Anesthetic Peripheral Nerve Block Adjuvants for Prolongation of Analgesia: A Systematic Qualitative Review

**DOI:** 10.1371/journal.pone.0137312

**Published:** 2015-09-10

**Authors:** Meghan A. Kirksey, Stephen C. Haskins, Jennifer Cheng, Spencer S. Liu

**Affiliations:** 1 Department of Anesthesiology, Hospital for Special Surgery, New York, New York, United States of America; 2 Department of Anesthesiology, Weill College of Medicine at Cornell University, New York, New York, United States of America; Eberhard Karls University, GERMANY

## Abstract

**Background:**

The use of peripheral nerve blocks for anesthesia and postoperative analgesia has increased significantly in recent years. Adjuvants are frequently added to local anesthetics to prolong analgesia following peripheral nerve blockade. Numerous randomized controlled trials and meta-analyses have examined the pros and cons of the use of various individual adjuvants.

**Objectives:**

To systematically review adjuvant-related randomized controlled trials and meta-analyses and provide clinical recommendations for the use of adjuvants in peripheral nerve blocks.

**Methods:**

Randomized controlled trials and meta-analyses that were published between 1990 and 2014 were included in the initial bibliographic search, which was conducted using Medline/PubMed, Cochrane Central Register of Controlled Trials, and EMBASE. Only studies that were published in English and listed block analgesic duration as an outcome were included. Trials that had already been published in the identified meta-analyses and included adjuvants not in widespread use and published without an Investigational New Drug application or equivalent status were excluded.

**Results:**

Sixty one novel clinical trials and meta-analyses were identified and included in this review. The clinical trials reported analgesic duration data for the following adjuvants: buprenorphine (6), morphine (6), fentanyl (10), epinephrine (3), clonidine (7), dexmedetomidine (7), dexamethasone (7), tramadol (8), and magnesium (4). Studies of perineural buprenorphine, clonidine, dexamethasone, dexmedetomidine, and magnesium most consistently demonstrated prolongation of peripheral nerve blocks.

**Conclusions:**

Buprenorphine, clonidine, dexamethasone, magnesium, and dexmedetomidine are promising agents for use in prolongation of local anesthetic peripheral nerve blocks, and further studies of safety and efficacy are merited. However, caution is recommended with use of any perineural adjuvant, as none have Food and Drug Administration approval, and concerns for side effects and potential toxicity persist.

## Introduction

The use of local anesthetic peripheral nerve blocks for surgical anesthesia and postoperative pain management has increased significantly with the advent of ultrasound-guided techniques. While catheter-based techniques allow for sustained pain management during the perioperative period, they can present challenges related to patient management, catheter displacement, and the potential for increased infection risk [1]. Numerous recent randomized controlled trials and meta-analyses have examined the pros and cons of the use of various individual adjuvants thought to potentially enhance local anesthetic peripheral nerve blockade. Recent animal safety and clinical observational work has also introduced the concept of “multimodal perineural analgesia (MMPNA)” whereby multiple agents with differing mechanisms of action are used with the goal of providing perineural analgesia while avoiding exposure to high and potentially toxic levels of individual agents [2–7].

The purpose of this systematic literature review is to provide clinical recommendations based on a comprehensive evaluation and summary of evidence for each commonly used and studied peripheral nerve block adjuvant that is used for the specific purpose of prolongation of postoperative analgesia provided by local anesthetic peripheral nerve blocks. While peripheral nerve block adjuvants are in widespread clinical off-label use and have been subject to multiple clinical trials, it should be noted that no adjuvant has been approved by the Food and Drug Administration (FDA) for prolongation of peripheral nerve blocks, and adjuvants should be used with appropriate caution.

## Materials and Methods

### Literature Search

**Fig 1:**
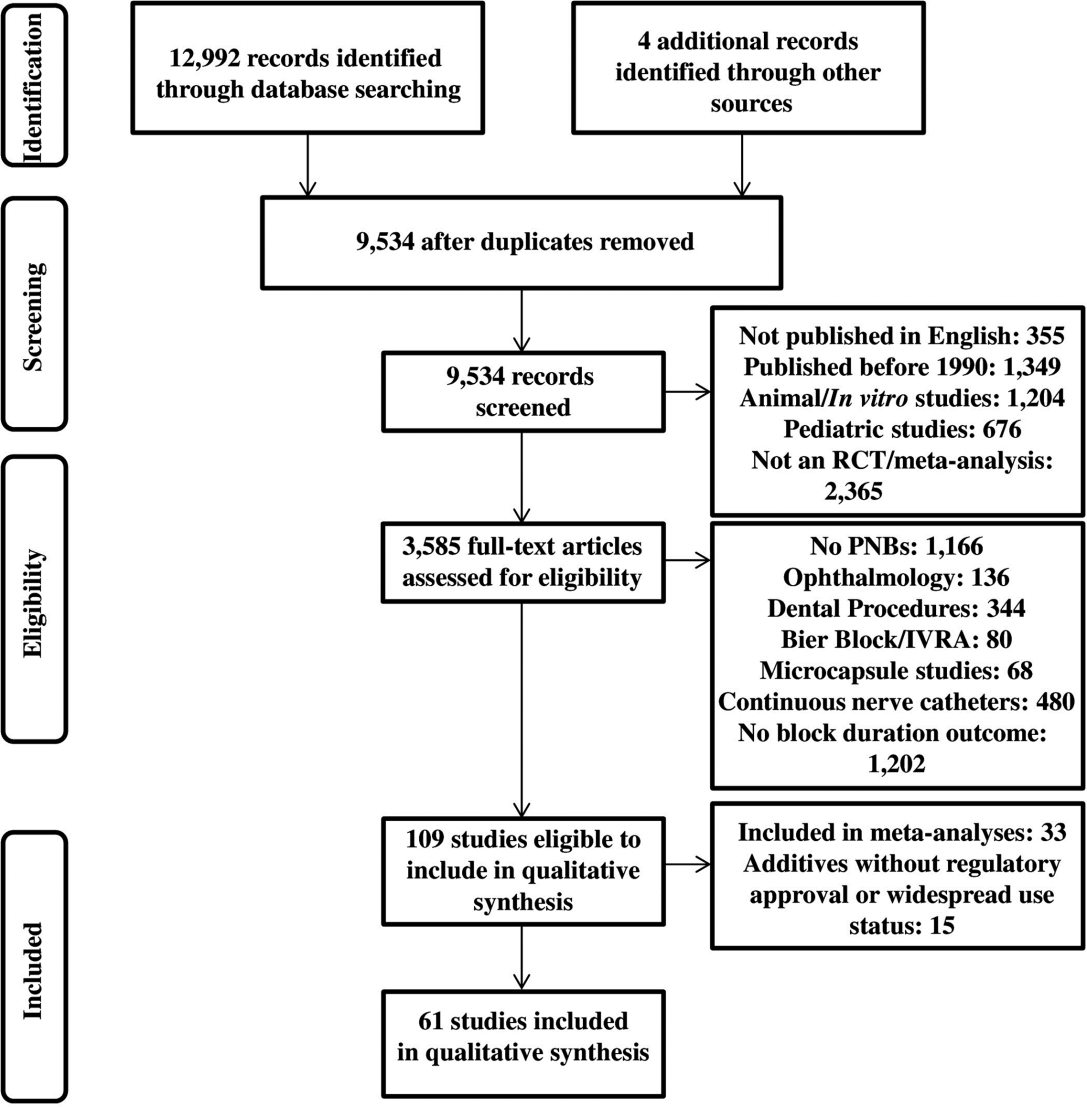
PRISMA flowchart. Details regarding records that were identified, screened, and assessed for eligibility are provided, according to the PRISMA guidelines.

A comprehensive search of the EMBASE, Medline/PubMed, and Cochrane Central Register of Controlled Trials bibliographical databases was performed for papers published between 1990 and 2014. Independent literature searches were performed by each of the four authors, and results were collated. The search terms used were *nerve block* ± *neurotoxicity* ± one of the following terms: *adjuncts*, *adjuvants*, *opioids*, *fentanyl*, *buprenorphine*, *morphine*, *tramadol*, *magnesium*, *vasoconstrictors*, *epinephrine*, *ketamine*, *non-steroidal anti-inflammatory drug (NSAID)*, *midazolam*, *parecoxib*, *ketorolac*, *clonidine*, *dexmedetomidine*, *dexamethasone*, *neostigmine*, *or potassium* with the definition exploded. Randomized controlled trials and meta-analyses of clinical trials published in English and including block and/or analgesia duration as an outcome were included. Authors were not contacted for supplemental data or information. Secondary references not included in the original search were derived from references of included trials and reviews. References for pediatrics studies, continuous nerve catheters, ophthalmologic blocks, microcapsule studies, dental blocks, and intravenous regional anesthesia/Bier blocks were excluded. Non-peer-reviewed papers, informal reviews, abstracts, letters, and opinion pieces were excluded. Individual references that were covered by included meta-analyses (Table 1) were excluded from this review. References to adjuvants not in widespread use and lacking Investigational New Drug application (IND) status or exemptions were excluded. The review protocol was not registered. The PRISMA flowchart is shown in Fig 1, and the PRISMA checklist is shown in S1 File.

**Table 1:** Summary of meta-analyses analyzing adjuvants for analgesia/block duration.

Author	Number of Trials	Adjuvant	Block	Minutes of Increased Duration	Side Effects
Choi et al. (2014)[72]	9	Dexamethasone	Brachial Plexus	Long-acting LA (730–1306 minutes) and Intermediate-acting LA (168–343 minutes)	None
Abdallah et al. (2013)[63]	4	Dexmedetomidine	Brachial Plexus	284 minutes (not reaching significance; p = 0.05)	Reversible bradycardia (7% of patients)
Popping et al. (2009)[51]	20	Clonidine	All (14/20 brachial plexus)	122 minutes	Arterial hypotension (OR 3.6), orthostatic hypotension/fainting (OR 5.1), bradycardia (OR 3.1), sedation (OR 2.3)

Abbreviations: LA = local anesthetic; OR = odds ratio

### Data extraction and assessment of study design quality

**Fig 2:**
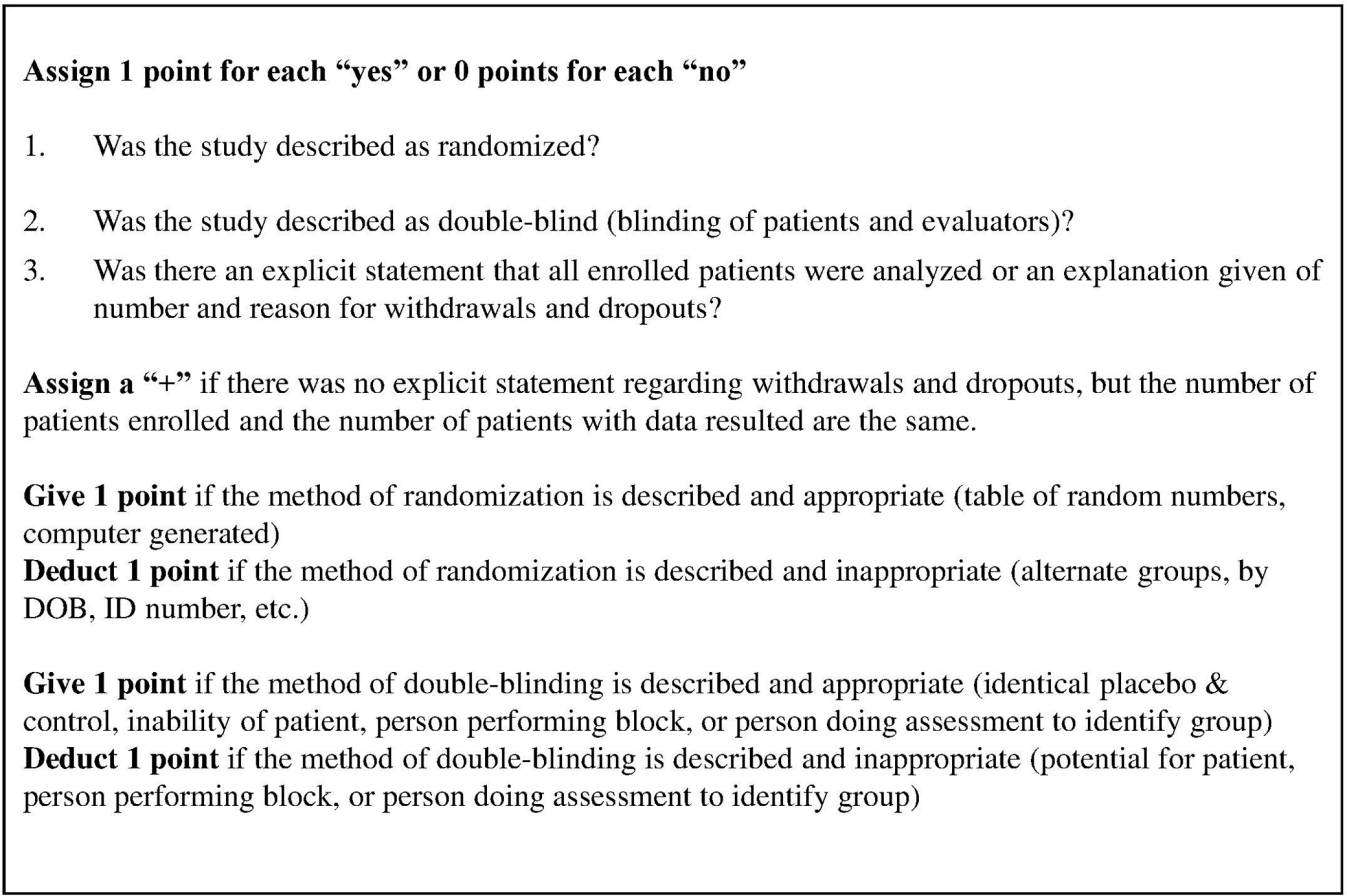
Modified Jadad Scale. Scoring guidelines for the modified Jadad scale, which was used to assess each study, are shown.

Data regarding study design, study population, interventions (peripheral nerve blocks with additives), and outcomes (block or analgesic duration) were extracted from each study that was included in the review. Study design quality and bias were assessed independently by at least two authors, and differing assessments were resolved by consensus of all authors. For each randomized controlled trial, study design quality was assessed independently by two authors (M.K. and S.H.) according to the Jadad scoring system [8]. The system was modified for this review to account for the subset of papers that report results for the full number of enrolled patients, but did not receive a point for an explicit statement about dropouts or withdrawals. Such studies were given a “+” status (Fig 2).

### Assessment of strength of evidence

The strength of evidence for each adjuvant was reported along with parameters of quality, quantity, consistency, and clinical significance of the published literature. These categories were designed based on the guidelines of the Evidence Strength Grid of the Agency for Healthcare Research and Quality evidence report “Systems to Rate the Strength of Scientific Evidence” [9]. Quality is reported as the fraction of studies with modified Jadad scores of III+ or greater. Consistency is recorded as the percent of studies with positive results. Clinical significance is described as low, moderate, or high based on the duration of block prolongation noted in the studies analyzed.

Grades of strength of recommendations are reported based on the Oxford Center for Evidence Based Medicine Levels of Evidence document [10]. Accordingly, Grade A recommendations are those based on Level 1 evidence (evidence from meta-analyses of clinical trials and/or at least one randomized controlled trial).

## Results

The bibliographic database search process and outcomes are summarized in Fig 1 in accordance with PRISMA guidelines. A total of 12,992 papers were found in the initial search and four from analysis of secondary sources. After duplicates were removed, 9,534 papers remained. Of these, 3,585 randomized controlled trials and systematic reviews of studies conducted on adult humans and published in English were screened for eligibility. Of the 109 studies meeting eligibility criteria, 33 had been analyzed previously in published meta-analyses referenced in this paper (Table 1). Fifteen studies were excluded because they involved agents that are not in widespread use or were performed without IND or equivalent status or exemption. A total of 61 novel clinical trials and meta-analyses were included in this systematic qualitative literature review.

A detailed summary of each randomized controlled trial is shown in Table 2, including: adjuvant dose, local anesthetic used, block performed, duration of analgesia prolongation, significant side effects potentially attributable to adjuvant, and study quality, as assessed by the modified Jadad score.

**Table 2:** Clinical findings for most extensively studied agents not covered by recent meta-analyses.

Agent	Local Anesthetic	Site/Dose	Prolongation of Analgesia or Sensory Block	Side Effects & Toxicity	Systemic Control (route)	Jadad Scale (I-V)
**Buprenorphine**	Bupivacaine 0.5% + epi[18]	Sciatic—0.3mg	6h*	PONV events: 7 in control group, 21 in IM buprenorphine group, 19 in PN buprenorphine group	No	V
**Buprenorphine**	Mepivacaine 1% + tetracaine 0.02% + epi[16]	Axillary—0.3mg	15h**	None	No	III+
**Buprenorphine**	Mepivacaine 1% + tetracaine 0.02% + epi[17]	SCB—0.3mg	12h?	PONV in 2/20 in PN buprenorphine group, 6/20 in IM buprenorphine group, and 3/20 in control group	Yes	V
**Buprenorphine**	Levobupivacaine 0.75%[19]	ISB—0.15mg	6h***	PONV in 4/50 pts; hypotension in 1/50 pts	No	IV+
**Buprenorphine**	Lidocaine 1% + bupivacaine 0.5%[15]	SCB- 3mcg/kg	9h*	Pruritus in 4/20 pts; PONV in 10/20 pts	No	II
**Buprenorphine**	Bupivacaine 0.3%[20]	SCB- 3mcg/kg	6h**	PONV in 2/20 pts in PN buprenorphine group and 2/20 pts in IM buprenorphine group. No buprenorphine-free control group.	Yes (IM)	III
**Morphine**	Bupivacaine 0.5% + epi[25]	ISB- 5mg	None	1) PONV in 5/20 pts in placebo group and 10/20 pts in morphine group. Pruritus in 3/20 in placebo group and 0/20 in morphine group.	No	III
**Morphine**	Bupivacaine 0.5%[26]	Intercostal- 4mg	None	None	No	III
**Morphine**	Lidocaine 1.5% + epi[24]	Axillary—0.1mg/kg	None (Note: decreased opiate consumption)	Pruritus in 1/20 PN morphine; nausea in 1/20 PN morphine and 2/20 IV morphine. No morphine-free control group.	Yes (IV)	III
**Morphine**	Lidocaine 1% + bupivacaine 0.5%[15]	SCB- 75mcg/kg	10h*	Pruritus in 1/20 pts. PONV in 2/20 in morphine group, 1/20 in control group	No	II
**Morphine**	Lidocaine 1% + bupivacaine 0.5%[27]	Axillary- 4mg	None	PONV in 2/19 PN morphine and 4/21 IV morphine. No morphine-free control group.	Yes (IM)	III
**Morphine**	Bupivacaine 0.125%[28]	Popliteal- 10mg	3h*	Somnolence and nausea in 14/46 pts in morphine phase and 0/46 pts during bupivacaine-alone phase. Decreased BP and HR described in morphine phase without data.	No	IV
**Fentanyl**	Ropivacaine 0.75%[30]	Axillary- 20mcg	None	Not reported	No	V
**Fentanyl**	Lidocaine 1.5% + epi[29]	Axillary- 100mcg	None	Not reported	No	V
**Fentanyl**	Lidocaine 1.5% + epi[31]	Axillary- 100mcg	1h**,***	Not reported	Yes (IV)	V
**Fentanyl**	Ropivacaine 0.75%[33]	Sciatic/femoral- 1mcg/kg	None	No difference in sedation or oxygen saturation	No	IV+
**Fentanyl**	Mepivacaine 1.5% + epi[32]	SCB- 75mcg	1h**	Not reported	Yes (IM)	III
**Fentanyl**	Lidocaine 1.5%[34]	ISB- 75mcg	None	Not reported	No	V
**Fentanyl**	Bupivacaine 0.25%[35]	Axillary- 100mcg	3h***, 10h**	Nausea in 1/20 pts in each fentanyl group, 0/20 in control group. No sedation in any group.	No	III+
**Fentanyl**	Articaine 2%[36]	Axillary- 100mcg	2h*, 1h***	5/22 with sedation in fentanyl group, 2/22 with sedation in control group	No	V
**Fentanyl**	Bupivacaine 0.25% + épi[37]	Paravertebral—0.6mcg/kg	12h*	None	No	V
**Fentanyl**	Bupivacaine 0.5% + lidocaine 2%[38]	Cervical plexus- 50mcg	3h*	Bradycardia in 2/38 in fentanyl group, 1/39 in control group. Hypercapnia in 3/38 in fentanyl group, 1/38 in control group.	No	V
**Epinephrine**	Lidocaine 1.5%[42]	Axillary- 200mcg/ml	45min**	Tachycardia and hypertension with 200mcg	No	IV
**Epinephrine**	Mepivacaine 1%[43]	Brachial plexus- 200mcg	1h***	None	No	III+
**Epinephrine**	Ropivacaine 0.5% and 0.2%[44]	Femoral- 5mcg/ml	None*	None	No	IV
**Clonidine**	Bupivacaine 0.375%[53]	Sciatic popliteal- 100mcg	~3-4h**	None	Yes (IM)	V
**Clonidine**	Levobupivacaine 0.5%[54]	Sciatic popliteal- 150mcg	None*	50% with clonidine experience. >20% decrease in systolic BP	No	V
**Clonidine**	Ropivacaine 0.5%[55]	Axillary- 150mcg	None***	None	No	IV+
**Clonidine**	Bupivacaine 0.5%[56]	SCB- 1mcg/kg vs. 2mcg/kg	21h with 2mcg/kg, 15h with 1mcg/kg	Higher hypotension, bradycardia, and sedation in 2mcg/kg group	No	V
**Clonidine**	Bupivacaine 0.5%[57]	SCB- 30mcg	220min*	Sedation	No	V
**Clonidine**	Lidocaine 1.5% (note: comparison to epinephrine 5mcg/ml)[58]	Cervical plexus- 5mcg/ml	None**	Increased lidocaine plasma concentrations compared to epinephrine	No	V
**Clonidine**	Bupivacaine 0.5% and lidocaine 2% (note: comparison to 5mg midazolam)[59]	SCB- 150mcg	None**	None	No	I+
**Dexmedetomidine**	Bupivacaine 0.375%[64]	SCB- 100mcg	~8h*	Bradycardia in one patient	No	III+
**Dexmedetomidine**	Ropivacaine 0.5%[65]	ISB- 150mcg	~4h**	Lower HR with dexmedetomidine, no neurotoxicity	No	V
**Dexmedetomidine**	Ropivacaine 0.375%[66]	Cervical plexus- 1mcg/kg	~50min**	Sedation, bradycardia requiring atropine	No	III+
**Dexmedetomidine**	Mepivacaine 1%[43]	Brachial plexus- 1mcg/kg	~75min**	Bradycardia	No	III+
**Dexmedetomidine**	Ropivacaine 0.75%[67]	Ulnar nerve block- 20mcg	~200min***	None	Yes (IV)	IV
**Dexmedetomidine**	Ropivacaine 0.5%[68]	Posterior tibial- 1mcg/kg	~4.5h**	Hypotension, bradycardia	No	V
**Dexmedetomidine**	Bupivacaine 0.25%[69]	SCB- 1mcg/kg	~180min*	Bradycardia	No	V
**Dexamethasone**	Lidocaine 1.5% + epi[73]	SCB- 8mg	3h**	None	No	V
**Dexamethasone**	Prilocaine 2%[76]	Axillary- 8mg	3h**	Not reported	No	IV+
**Dexamethasone**	Bupivacaine 0.5%[77]	Sciatic/saph- 8mg; ankle- 8mg	Sciatic/saph—13% of patients with pain in first 24hrs vs. 47% in IM group; ankle—none	Not reported	Yes (IM)	V
**Dexamethasone**	Ropivacaine 0.5%[78]	ISB- 10mg	None	3.8- and 5.1-mg/dL increase in blood glucose with PN and IV administration	Yes (IV)	V
**Dexamethasone**	Bupivacaine 0.5% + epi[79]	Sciatic- 8mg	None	Statistically insignificant increase in incidence of numbness and paresthesia at 24 and 48hrs. No symptoms persisted at 8wks in any group.	Yes (IV)	V
**Dexamethasone**	Bupivacaine 0.25%[74]	TAP- 8mg	1h*	Decreased nausea and vomiting (6/30 with dexamethasone vs. 14/30 with control).	No	IV+
**Dexamethasone**	Bupivacaine 0.25%[81]	SCB- 1mg, 2mg, 4mg	10h*	One transient paresthesia noted in 2mg group	Yes (IV)	V
**Tramadol**	Levobupivacaine 0.5%[89]	ISB—1.5mg/kg	7h*	None	Yes (IM)	V
**Tramadol**	Lidocaine 1.5% + epi[90]	Axillary- 200mg	160min*, 65min***	None	No	V
**Tramadol**	Levobupivacaine 0.5% + lidocaine 2%[91]	Axillary- 100mg	None*	Sedation, nausea	No	IV+
**Tramadol**	Levobupivacaine 0.5%[92]	Psoas—1.5mg/kg	None*	None	Yes (IV)	V
**Tramadol**	Mepivacaine 1.5%[93]	Axillary- 40mg, 100mg, 200mg	60min, 40min, 40min*	Nausea/vomiting	No	IV
**Tramadol**	Mepivacaine 1%[94]	Axillary- 100mg	100min***	None	Yes (IV)	IV
**Tramadol**	Ropivacaine 0.75%[95]	Axillary- 100mg	None*	None	No	V
**Tramadol**	Bupivacaine 0.5%[96]	Paravertebral—1.5mg/kg	None*	None	No	V
**Magnesium**	Bupivacaine 0.25%[102]	Femoral- 500mg	10h*, 2h***	Not reported	No	III
**Magnesium**	Bupivacaine 0.5%[103]	ISB- 200mg	2h*	Nausea 2-3x more frequently at 4, 8, and 12hrs postoperatively with magnesium	No	V
**Magnesium**	Prilocaine 2%[104]	Axillary- 150mg, 100mg	2h***, 1h***	None	Yes (150mg IV)	II
**Magnesium**	Levobupivacaine 0.5%[105]	Axillary- 150mg	150min***	No thrombi or vasospasm in any group. Other side effects not reported.	No	III+
**Magnesium**	Levobupivacaine 0.25%[105]	Axillary- 150mg	100min***	No thrombi or vasospasm in any group. Other side effects not reported.	No	III+

Abbreviations: SCB = supraclavicular block; ISB = interscalene block; PN = perineural; IM = intramuscular; IV = intravenous; BP = blood pressure; HR = heart rate; saph = saphenous; POD = postoperative day; SQ = subcutaneous; GI = gastrointestinal; TAP = transversus abdominis plane; epi = epinephrine; PONV = postoperative nausea and vomiting; pts: patients.

*Time to first analgesic;

**Time to first reported pain;

***Time to pinprick or restoration of sensation.

### Opioids

#### Buprenorphine

The discovery of peripheral opioid receptors led to the clinical application of adding opioids to local anesthetics for peripheral nerve blocks [11]. Buprenorphine is a highly lipophilic partial opioid receptor agonist which may also have the local-anesthetic-like capacity to block voltage gated Na^+^ channels [12]. Buprenorphine has been shown to have notable antihyperalgesic effects, which may be attributable in part to the fact that buprenorphine and its metabolite norbuprenorphine have been shown to act on κ and δ opioid receptors in addition to μ receptors [13, 14].

In 1997, Bazin et al. showed that buprenorphine prolonged the median duration of analgesia from combination lidocaine/bupivacaine supraclavicular blocks from 11.5 to 20 hours [15]. Studies by Candido et al. demonstrated that buprenorphine induced 1.5- to 3-fold prolongation of subclavian [16] and axillary [17] blocks performed with a mixture of mepivacaine and tetracaine plus epinephrine and sciatic nerve blocks performed with bupivacaine plus epinephrine [18]. Similarly, Behr et al. demonstrated a near doubling of the time to first analgesic request when perineural buprenorphine was added to levobupivacaine interscalene blocks for shoulder arthroscopy [19]. Jadon et al. established that bupivacaine supraclavicular blocks with perineural buprenorphine were prolonged by nearly 6 hours compared to bupivacaine blocks with intramuscular buprenorphine [20]. None of these studies detected a difference in respiratory depression, nausea, or vomiting between those receiving buprenorphine and those receiving local anesthetic without opioids. It should be noted, however, that in several studies, the risk of postoperative nausea and vomiting (PONV) was found to be elevated with the use of perineural buprenorphine (Table 2).

Buprenorphine has been consistently shown to significantly prolong peripheral nerve blocks. However, we recommend that it be used only with multimodal nausea prophylaxis with agents such as perphenazine [21–23], dexamethasone, and a 5-HT3 antagonist [21, 22]. Consideration should be given to avoiding use of buprenorphine in patients with a significant history of PONV, pending clinical studies confirming that multimodal antinausea prophylaxis can adequately prevent this side effect in this patient population.

#### Morphine

Studies from the 1990s showed mixed results from the addition of morphine to peripheral nerve blocks, with two suggesting enhancement of analgesia duration [15, 24] and several showing no benefit at all [25–27]. Bazin et al. found that the addition of morphine to combination lidocaine/bupivacaine supraclavicular blocks prolonged the median duration of analgesia after internal fixation of upper extremity fractures from 11.5 to 21 hours [15]. Bourke et al. failed to detect a difference in postoperative visual analog scores, motor, or sensory block duration with perineural morphine when added to axillary blocks performed with lidocaine plus epinephrine when compared to intravenous (IV) morphine supplementation [24]. However, they did report a significantly decreased consumption of supplemental opioid doses. Flory et al. found that morphine did not prolong interscalene blocks performed with bupivacaine plus epinephrine for elective shoulder surgery [25]. Sternlo et al. were not able to show prolongation of 0.5% bupivacaine intercostal blocks with the addition of morphine [26]. Racz et al. showed that analgesia duration with intramuscular (IM) morphine was indistinguishable from morphine mixed with local anesthetic after hand and forearm surgery with mixed lidocaine/bupivacaine axillary blocks [27].

Keskinbora et al. recently examined the use of bupivacaine versus bupivacaine plus morphine administered via a popliteal catheter for patients with chronic lower extremity pain. The study included a short-term single bolus treatment phase where morphine was noted to prolong analgesia by approximately three hours compared to bupivacaine alone [28]. It should be noted, however, that the morphine group experienced significantly greater side effects (nausea and somnolence). More than twice as many patients preferred treatment with bupivacaine alone, even though they required more rescue analgesia with lornoxicam.

Overall, evidence indicates that the benefits of perineurally administered morphine, if any, do not surpass those of IV or IM morphine and may be outweighed by side effects, such as pruritus, PONV, and somnolence (Table 2). The routine use of perineural morphine is not recommended.

#### Fentanyl

In 2000, Nishikawa et al. found that the addition of fentanyl to axillary blocks with lidocaine plus epinephrine increased block duration by approximately one hour, but delayed block onset in all branches. It was speculated that this delay was caused by differences in pH of the injectates [29]. Multiple other papers have failed to show a benefit from perineural use of fentanyl as an adjuvant to local anesthetics for prolongation of peripheral nerve blocks. Fentanyl failed to prolong duration of axillary blocks with ropivacaine [30] or lidocaine plus epinephrine [31], supraclavicular blocks with mepivacaine plus epinephrine [32], sciatic/femoral blocks with ropivacaine [33], and interscalene blocks with lidocaine [34].

Several more recent publications have rekindled the interest in perineural administration of fentanyl. Karakaya et al. and Sert et al. demonstrated prolongation of motor and sensory block as well as statistically significant increases in analgesic duration when fentanyl was added to bupivacaine [35] or articaine [36] axillary blocks without epinephrine. Bhuvaneswari et al. found that addition of fentanyl to 0.25% bupivacaine plus epinephrine paravertebral blocks prolonged analgesia to 18 hours, which was comparable to the duration of blocks with 0.5% bupivacaine plus epinephrine [37]. Finally, Sindjelic et al. were recently able to show an improvement in quality and duration of mixed bupivacaine/lidocaine cervical plexus blocks for carotid endarterectomy surgery [38].

Small increases in rates of sedation, bradycardia, and hypercapnia have been seen in some trials when fentanyl was added to peripheral nerve blocks (Table 2). While it is possible that fentanyl may be effective for prolonging bupivacaine peripheral nerve blocks, the evidence regarding its efficacy is conflicting. We do not recommend perineural fentanyl for routine use.

#### Other Opioids

The interest in the potential for opioids to enhance peripheral nerve blocks has led to preliminary studies of several other agents. This review found no such studies, however, that were performed with IND or equivalent status or exemption. While the use of these agents merits further preclinical and clinical studies with appropriate approval, they cannot currently be recommended.

#### Toxicity and Side Effects

A 1997 review of peripherally administered opioids, which included 26 randomized controlled trials and 952 patients, 485 of whom received opioids, noted no adverse effects in any of the trials which could be attributed to the route of administration [11]. It should be noted, however, that many trials examining peripheral administration of opioids reported side effects typical of systemic administration, including pruritus, nausea, and vomiting. Sabbe et al. found that intrathecal administration of sufentanil, fentanyl, and morphine to dogs led to no histological signs of neurotoxicity after 28 days of daily exposure [39].

In contrast, *in vitro* studies have shown some signs of neurotoxic potential of opioids. Although sufentanil and morphine did not enhance lidocaine-induced cell death in human neuroblastoma cells, morphine increased lidocaine-induced apoptosis of rat astrocytes, whereas sufentanil did not [40]. Perineural buprenorphine has consistently shown the ability to prolong peripheral nerve blocks with no reported increase in side effects or clinical toxicity and may be considered a useful adjuvant for block prolongation. It should be noted, however, that in studies of isolated rat primary sensory neurons, high-concentration buprenorphine exposure for 24 hours results in significant cell death [2]. Further laboratory analysis of neuronal exposure to clinically relevant concentrations of buprenorphine in isolation and in combination with local anesthetics and other perineural analgesic adjuvants is warranted.

### Vasoactive Agents

#### Epinephrine

Epinephrine has been used for over a century as an additive to local anesthetics [41]. With a typical dose range of 5–10mcg/mL, epinephrine is believed to prolong duration by its vasoconstrictive properties that prevent systemic reabsorption of local anesthetics. Epinephrine can be added to local anesthetics to detect intravascular injection, and its vasoconstrictive properties have the presumed added benefit of decreasing systemic toxicity, allowing for larger doses of local anesthetic to be given safely. Epinephrine has shown mixed efficacy as an adjuvant to prolong nerve blockade (Xylocaine [lidocaine hydrochloride] package insert; Schaumberg, IL: APP Pharmaceuticals, LLC, 2010). Using lidocaine with high-dose epinephrine (200mcg/mL) for axillary block prolonged motor block and sensory block by approximately 25 and 40 minutes, respectively, but was associated with tachycardia and hypertension. A lower dose of 25mcg/mL had minimal effect, prolonging motor block by 10 minutes and sensory block by 30 minutes [42]. When added to mepivacaine for brachial plexus block, epinephrine prolonged motor and sensory block duration by approximately 60 minutes [43]. Epinephrine has not been shown to prolong blockade with ropivacaine [2, 44] (Naropin [ropivacaine hydrochloride] package insert; Schaumberg, IL: APP Pharmaceuticals, LLC, 2010) and is an inferior additive when compared to other adjuvants, such as clonidine, in prolonging brachial plexus block with bupivacaine [45].

Despite its long-term use as an adjuvant in local anesthetics, epinephrine has been shown to compromise endoneural blood flow [46] and increase neurotoxicity, particularly in the setting of diabetic animal models, arguing against its use in patients with diabetic peripheral neuropathy [3, 47, 48]. Epinephrine has been shown to have minimal efficacy in prolongation of peripheral nerve blocks, and we do not recommend its use for this purpose. When epinephrine is used to detect intravascular injection, caution is merited, as it can cause hypertension and tachycardia in high doses and may exacerbate neurovascular compromise in susceptible patients.

#### Clonidine

Clonidine hydrochloride is an alpha-2 agonist that has vasoconstrictor properties, but, unlike epinephrine, its ability to prolong nerve blockade is due to direct action on peripheral nerves. Recent data suggest that this prolongation is mediated by hyperpolarization of cyclic-nucleotide-gated cation channels [49]. Clonidine was first described as an epidural additive in 1984 [50], and multiple subsequent randomized controlled trials and several meta-analyses have demonstrated the utility of clonidine as an additive to prolong peripheral nerve blockade, particularly when used with intermediate- to long-acting local anesthetics. A meta-analysis by Popping et al. reviewed 20 studies and found that clonidine extended average block duration by approximately 2 hours [51]. A qualitative review by McCartney et al. analyzed 27 studies and found mixed results regarding clonidine’s ability to prolong nerve blockade [52]. This review cited 15 positive studies and 12 negative studies and suggested that clonidine prolongs nerve blockade best when added to intermediate-acting local anesthetics, particularly mepivacaine and lidocaine (with less support for bupivacaine and levobupivacaine). Both Popping and McCartney showed that increasing doses of clonidine resulted in an increased incidence of systemic absorption, thereby causing hemodynamic side effects including hypotension, bradycardia, and fainting. Both authors ultimately recommended dosing clonidine at 0.5mcg/kg with a maximum dose of 150mcg.

Since the publication of these reviews, there continues to be conflicting experimental data with perineural clonidine. Clonidine was shown to prolong bupivacaine popliteal sciatic nerve block by approximately 3–4 hours with 0.375% bupivacaine [53] but did not prolong blockade with 0.5% levobupivacaine [54]. A recent study showed no benefit of adding 150mcg of clonidine to ropivacaine for axillary block [55], whereas another demonstrated a notably faster onset and longer duration of action of analgesia with high-dose clonidine (2mcg/kg) with no “major” hemodynamic side effects when added to bupivacaine for supraclavicular block [56]. Another positive study showed that addition of clonidine to bupivacaine prolonged duration of supraclavicular block by approximately 200 minutes [57]. In contrast, when compared to 5 mcg/ml epinephrine, clonidine led to no difference in block duration when added to lidocaine for cervical plexus block and resulted in a higher plasma concentration of lidocaine [58]. Another study failed to detect a difference in block duration between clonidine and midazolam when combined with bupivacaine plus lidocaine for supraclavicular block [59].

Despite substantial study, it is not clear which local anesthetics, anatomical blocks, or doses of clonidine are optimal for prolongation of analgesia after peripheral nerve blocks. It is clear, however, that high doses result in systemic side effects, such as hypotension and bradycardia, and should be avoided. The use of perineural clonidine merits further high quality preclinical and clinical study but is not currently recommended for routine use.

#### Dexmedetomidine

Dexmedetomidine is an alpha-2 agonist with seven times greater affinity than that of clonidine [60]. Much like clonidine, dexmedetomidine has been shown in a rat model to prolong duration of analgesia by blocking the hyperpolarization-activated cation current [61]. Dexmedetomidine was first used as an additive in 2004 to supplement intravenous regional anesthesia [62]. Multiple randomized controlled trials have since been conducted, and a recent meta-analysis was performed to examine its effectiveness as a peripheral nerve block additive [63]. Abdallah et al. recently published a meta-analysis that examined four studies of dexmedetomidine as an additive for brachial plexus blocks [63]. This analysis found that dexmedetomidine significantly prolonged mean motor block by 268 minutes and time to first analgesic by 345 minutes. However, the mean sensory block prolongation of 284 minutes was not statistically significant. The doses in the four studies looking at brachial plexus blocks were 30mcg, 100mcg, 0.75mcg/kg, and 1mcg/kg. None of the studies examined in this review described hypotension as an adverse effect, and reversible bradycardia was seen less than 10% of the time.

Two more recent studies have shown that the addition of dexmedetomidine to bupivacaine supraclavicular blocks and ropivacaine interscalene blocks prolonged the duration of the blocks by approximately 8 hours [64] and 4 hours, respectively [65]. The first of these studies noted one episode of bradycardia, and both studies demonstrated improved postoperative analgesia. Another recent study found that the addition of dexmedetomidine to ropivacaine for cervical plexus block increased block duration by approximately 50 minutes [66]. Similarly, a study looking at the addition of dexmedetomidine to mepivacaine for brachial plexus blocks showed a block prolongation of approximately 75 minutes; however, the duration was minimally increased when compared to 200mcg epinephrine [43]. Volunteer studies have also demonstrated the efficacy of dexmedetomidine. In one volunteer study, dexmedetomidine was added to ropivacaine ulnar nerve blocks and resulted in a 200-minute prolongation of analgesia [67]. In contrast, systemic dexmedetomidine increased the duration of analgesia by only 50 minutes. In another volunteer study, dexmedetomidine was added to ropivacaine for posterior tibial nerve blocks, resulting in a prolongation of analgesia by approximately five hours [68]. Of note, a recent study found that the duration of sensory and motor block when dexmedetomidine was added to bupivacaine supraclavicular blocks was almost twice as long compared to the addition of clonidine [69].

The potential for dexmedetomidine to cause neurotoxicity in humans has not been extensively studied. However, in animal models of spinal anesthesia [70] and sciatic nerve block [71], dexmedetomidine did not show toxicity and was potentially neuroprotective when combined with lidocaine [70] and bupivacaine [71]. The bulk of published data supports the efficacy of dexmedetomidine for peripheral nerve block prolongation of approximately 200 minutes at doses around 1mcg/kg, and it appears to be a viable option as an additive to ropivacaine or bupivacaine in patients where bradycardia and hypotension are likely to be treatable with conventional therapies.

### Anti-inflammatory Agents

#### Dexamethasone

A recent meta-analysis of randomized placebo-controlled trials through April 2013 was conducted by Choi et al. [72] and included 9 trials, with 393 patients receiving dexamethasone. This study concluded that dexamethasone prolongs brachial plexus blocks with long-acting local anesthetics from 730 to 1306 minutes and intermediate-acting anesthetics from 168 to 343 minutes. Several more studies of perineural dexamethasone have been published since this meta-analysis was performed. Brachial plexus blocks with lidocaine plus epinephrine were prolonged two-fold by the addition of dexamethasone in one recent study of patients undergoing elective hand, forearm, and elbow surgery [73]. Time to first analgesic request after transversus abdominis plane (TAP) blocks performed with bupivacaine for abdominal hysterectomy were prolonged by approximately one hour by the addition of dexamethasone [74]. Rasmussen et al. recently performed a retrospective review of 1,040 patient records and found that when added to ropivacaine, dexamethasone prolonged a range of upper and lower extremity peripheral nerve blocks by a median of 37% [75]. Of note, in this study, patients receiving dexamethasone reported statistically significantly decreased pain on the day of surgery, less severe postoperative pain, better satisfaction, and no increase in adverse events. A recent study by Saritas et al. demonstrated significant prolongation of prilocaine brachial plexus block with addition of dexamethasone; however, block duration remained shorter than in the group receiving levobupivacaine alone [76]. None of the above studies captured significant side effects associated with perineural steroid administration; however, decreased rates of PONV were noted when dexamethasone was used in TAP blocks for hysterectomy [74].

It should be noted that analgesia duration and block duration are not the same and that the analgesic impact of dexamethasone may be related to systemic effects. Fredrickson et al. found only a modest reduction in pain reported at 24 hours for patients who received bupivacaine sciatic/saphenous blocks with perineural dexamethasone versus subcutaneous/intramuscular dexamethasone [77]. No differences in onset of pain were found with a similarly protocolized ankle block study reported in the same paper. Two other recent randomized placebo-controlled studies showed no significant difference in analgesia duration when interscalene blocks with ropivacaine [78] and sciatic nerve blocks with bupivacaine plus epinephrine [79] were supplemented with 8-10mg dexamethasone intravenously versus perineurally. It is worth noting that intention to treat analysis was not strictly adhered to in the first study [78], and the question remains whether these studies were adequately powered to detect a clinically significant difference between administration routes [80].

Although perineurally administered dexamethasone has consistently been shown to prolong analgesia after peripheral nerve blocks, it is not clear that this finding is not due to systemic effects. A recent study by Liu et al. demonstrated that dexamethasone prolonged analgesia by approximately 10 hours compared to a control group for ambulatory shoulder surgery using 0.25% bupivacaine, and this was achieved with perineural doses ranging from 1mg, 2mg, and 4mg of preservative-free dexamethasone [81]. The effect seen by Liu et al. was despite the fact that most patients, including those in the control group, received IV dexamethasone as an antiemetic. One excluded observational study by Williams et al. reported an associated reduction of analgesic duration and elevation of rebound pain with 2mg (vs. 1mg) of perineural dexamethasone, with the 1–2mg perineural dexamethasone doses being much lower than all the doses cited above. Future research will need to better identify perineural dose-response of dexamethasone in the 1–2mg range (versus 4mg and higher) [5]. There is a theoretical risk of dexamethasone-induced peripheral neurotoxicity based on *in vitro* studies [2, 82, 83], and this must be weighed against the apparent efficacy of systemic administration. Further high-quality preclinical and clinical study is merited before it can be recommended for routine use.

#### Other Anti-inflammatory Agents

There are no studies of other widely used or novel anti-inflammatory agents that have received or been exempted from IND or equivalent status. Of note, a toxicity study of epidural administration of parecoxib in rats showed no neurologic behavioral or histological evidence of neurotoxicity [84]. However, lornoxicam, another NSAID, was shown to have dose-dependent neurotoxic effects after epidural administration to rabbits [85]. The perineural use of anti-inflammatory medications other than dexamethasone merits further pre-clinical and clinical study with appropriate federal approval; however, their use cannot be recommended at this time.

### Other Agents

**Tramadol** is a weak central-acting opioid that has been shown to have Na^+^ and K^+^ channel-blocking properties and can block motor and nociceptive function similarly to local anesthetics [86, 87]. There is conflicting data regarding the effect of adding tramadol to local anesthetics for peripheral nerve blocks. This topic was recently covered thoroughly in a review by Bailard et al. [88]. One study showed a doubling of analgesia duration after interscalene blocks for total shoulder arthroplasty when tramadol was added to levobupivacaine [89]. A study of high-dose tramadol added to lidocaine plus epinephrine showed an increase in sensory block duration of approximately two hours and time to first analgesic by approximately five hours [90]. In contrast, multiple publications have shown limited benefit of tramadol added to levobupivacaine plus lidocaine [91], levobupivacaine alone [92], and mepivacaine [93, 94], and no effect when added to ropivacaine [95] and bupivacaine [96]. Conflicting data were also noted from three studies comparing perineural tramadol to systemic tramadol. Kapral et al. found that adding tramadol to mepivacaine significantly prolonged sensory and motor axillary block when compared with mepivacaine plus IV tramadol [94]. Alemanno et al. showed that duration of analgesia from interscalene blocks with levobupivacaine plus tramadol was significantly longer than that from levobupivacaine plus IM tramadol [89]. However, Mannion et al. found that block duration, time to first analgesic, and total 24-hour opioid use were similar after psoas compartment blocks with levobupivacaine with or without IV or perineural tramadol [92].

Since little is known about the potential neurotoxicity of perineural tramadol, and the bulk of published literature shows little to no efficacy in prolongation of analgesia, we do not recommend its use.

**Midazolam** is a water-soluble benzodiazepine that is an indirect gamma-aminobutyric acid receptor agonist that has been studied primarily as an adjuvant for neuraxial anesthesia [97]. This agent is not FDA approved and is not routinely used as a perineural adjuvant. No clinical studies have been published after IND approval or exemption or its equivalent. Moreover, in animal models, intrathecal midazolam has been shown to be neurotoxic [98–100]. This agent should be avoided as a perineural adjuvant to local anesthetics.

**Magnesium** is an N-Methyl-D-aspartate (NMDA) antagonist that plays a role in moderating calcium influx into neurons. Magnesium has been shown to decrease peripheral nerve excitability and to enhance the ability of lidocaine to raise the excitation threshold of A-beta fibers [101]. Studies have consistently shown that addition of magnesium to local anesthetic significantly prolongs peripheral nerve blocks, including femoral nerve blocks with bupivacaine [102], interscalene blocks with bupivacaine [103], and axillary blocks with prilocaine [104] and levobupivacaine [105]. All of these papers denied adjuvant-related toxicity or side effects; however, nausea was two to three times more likely in the first 12 hours after interscalene blocks with 200mg magnesium in the study by Lee et al. [103]. This side effect was not reported in studies using 150mg magnesium. Notably, one animal study has shown histological evidence of nerve damage from intrathecal administration of magnesium [106]. However, a recent meta-analysis describes four trials that systematically recorded data on complications after intrathecal administration of magnesium [107]. In this analysis, the only complication noted was one patient out of 140 who experienced a four-day headache.

Magnesium appears to reliably prolong peripheral nerve blocks; however, potential neurotoxicity and side effects of peripherally administered magnesium have not been adequately studied for it to be recommended for use. Further high-quality pre-clinical and clinical studies are warranted. Specifically, animal studies are needed to determine if magnesium results in neurotoxicity with perineural administration when administered alone, in combination with local anesthetics, and as a component of multimodal drug combinations. Moreover, it remains to be seen if clinical efficacy can be achieved with lower doses of magnesium while avoiding the side effects of nausea and vomiting.

**Ketamine** is an NMDA receptor antagonist that has been shown to have local anesthetic properties [108]. There is limited data on the effect of ketamine as a perineural additive, and no studies with IND or equivalent approval or exemption were found in this review. It should be noted that one study, which met exclusion criteria, reported a high incidence (44%) of adverse effects such as hallucinations, drowsiness, and nausea with no prolongation of analgesia [109]. There are limited data on the peripheral neurotoxicity of ketamine. However, a recent study showed no histological signs of neurotoxicity after multiple intrathecal injections of preservative-free ketamine into rabbits [110]. Given the dearth of high-quality clinical data supporting its efficacy and the concern for high incidence of side effects with the use of perineural ketamine, we do not recommend its use or further clinical study as a perineural adjuvant.

**Neostigmine** is an acetylcholinesterase inhibitor that can enhance analgesia by increasing endogenous acetylcholine at the nerve terminal. No studies of neostigmine that were performed with an IND or equivalent approval were found. It is notable, however, that two excluded studies found that neostigmine failed to increase block duration but was associated with a relatively high incidence of nausea and associated gastrointestinal (GI) distress. In addition, Demirel et al. studied both intrathecal midazolam and neostigmine in a rabbit model and found a similar level of neurotoxicity [100]. In contrast, neostigmine did not increase human neuroblastoma or rat astrocyte cell death compared to lidocaine alone in an *in vitro* model [40]. Given the high incidence of GI side effects, risk for neurotoxicity and minimal data supporting block prolongation, we do not recommend the use or further study of neostigmine as a perineural adjuvant.

## Discussion

Concern for reporting bias in favor of publication of positive results must be acknowledged in this and other reviews of the published literature. However, the results presented in this study favor the efficacy of some agents over others, and publication bias is unlikely to favor one peripheral nerve block mono-adjuvant over another. In this study, risk of bias within studies was quantitated using a modified Jadad score to assess for selection, performance, attrition, and detection bias. Using a modified Jadad score cutoff of III+ or higher on a 5-point scale, 47 out of 60 included studies had a low risk of bias and were considered of high quality when determining recommendations. Among particular adjuvants, studies of morphine and magnesium were notable for having the highest percentage of studies with low modified Jadad score. It should be noted that most studies of these two agents predate current publication standards specifying randomization techniques and blinding methodology, both of which weigh heavily into the Jadad score. The summary of findings found in Table 3 includes a report of the strength of evidence, as assessed by (1) study quality as defined above; (2) quantity of studies; (3) consistency (based on the percentage of positive results); and (4) clinical significance (based on degree of block prolongation described).

**Table 3:** Summary of findings and recommendations.

Agent	Criteria for Inclusion^1^	Strength of Study Evidence^2^: a-Quality/Quantity; b-Consistency; c-Significance	Summary/Recommendations	Grade of Recommendation (level of evidence)^3^
Buprenorphine	Attestation	a- 4/6; b- 100%; c- high (all ≥ 6h)	Buprenorphine can significantly prolong PNB. Concern for PONV merits multimodal antinausea prophylaxis.	A (1b)
Morphine	FDA	a- 1/6; b- 33%; c- moderate (3h and 10h)	Not recommended due to lack of quality studies and lack of consistently positive results.	A (1b)
Fentanyl	Attestation	a- 9/10; b- 60%; c- moderate (3–12h for bupivacaine blocks)	May prolong bupivacaine PNB. Not recommended due to inconsistent results and concern for increased rates of sedation, bradycardia, and hypercapnia.	A (1b)
Epinephrine	Attestation	a- 3/3; b- 66%; c- low (no more than 1h)	May prolong blockade by a minimal amount (45–60min). High doses can result in systemic absorption, tachycardia, and hypertension. Avoid use in patients with preexisting neurovascular compromise, such as diabetic neuropathy.	A (1b)
Clonidine	Attestation	a- 6/7; b- 43%; c- moderate (3–6h for bupivacaine blocks)	Prolongs blockade with bupivacaine but does not appear to be effective with ropivacaine or levobupivacaine. *Meta-analysis of 20 other papers shows ~2-h prolongation of nerve block. High doses (2mcg/kg) can cause hypotension, bradycardia, and sedation via systemic absorption.	A (1a, 1b)
Dexmedetomidine	IND; Attestation	a- 7/7; b- 100%; c- moderate (1–8h)	Evidence supports block prolongation from 1–8h depending on the block and local anesthetic. *Meta-analysis of 4 other papers shows prolongation, but was not statistically significant. May increase bradycardia and sedation intraoperatively.	A (1a, 1b)
Dexamethasone	IND; Attestation	a- 6/6; b- 50% (100% with placebo control, 0% with systemic control); c- moderate (1–3h)	Perineural dexamethasone likely prolongs nerve blockade; however, analgesic effect is similar with systemic dexamethasone. Its use may decrease rates of PONV in procedures with high incidence. *Meta-analysis of 9 other papers supports prolongation of brachial plexus blocks compared to dexamethasone-free controls.	A (1a, 1b)
Tramadol	Attestation	a- 8/8; b- 50%; c- low with axillary (40–160min, 3 studies); high with ISB (7h, 1 study).	7/8 studies showed minimal to no prolongation of analgesia or nerve blockade. Not recommended due to lack of evidence of clinically significant efficacy and potential to increase sedation and PONV.	A (1b)
Magnesium	Attestation	a- 3/5; b- 100%; c- low for brachial plexus (1–2.5h, 4 studies); high for FNB (10h for analgesic request, 1 study)	Consistently shown to prolong PNB but likely not clinically significant for brachial plexus blocks. One study of moderate quality (Jadad III) suggests significantly increased duration of analgesia for FNB. Further high-quality studies needed to determine toxicity profile and minimal effective dose. Concern for PONV at 200mg dose. Not recommended at this time.	A (1b)
Ketamine	Harm	a- 2/2; b- 0%; c- N/A	Not recommended due to lack of evidence of efficacy and significant side effect profile (hallucinations, drowsiness, and nausea).	A (1b)
Neostigmine	Harm	a- 3/4; b- 25%; c- low (<1h)	Not recommended due to lack of evidence of efficacy, significant neurotoxicity in rabbit model, and high rate of GI distress.	A (1b)
Midazolam	Harm	a- 0/2; b- 100%; c- moderate (3h)	Not recommended due to established neurotoxicity when administered with local anesthetics in animal models, high incidence of sedation, and lack of quality clinical studies.	A (1b)

^1^Attestation: Referenced in textbooks and/or multiple (>5) peer-reviewed research publications; Harm: Not Food and Drug Administration (FDA)-approved and balance of evidence suggests harm with perineural use; FDA: FDA-approved for regional anesthesia; IND: Investigational New Drug status (or international equivalent) granted or waived for in at least one reviewed study.

^2^a: Studies with Jadad score III+ or higher/total number of studies; b: % of studies with positive results; c: clinical significance of positive results (extent of prolongation of analgesia or sensory block).

^3^Agency for HealthCare Research and Quality Levels of Evidence and Grades of Recommendations: Grade A: based directly on Level 1 evidence; Level 1a: evidence from meta-analysis of clinical trials; Level 1b: evidence from at least 1 randomized controlled trial.

Abbreviations: PNB = peripheral nerve block; PONV = postoperative nausea and vomiting; ISB = interscalene block; FNB = femoral nerve block; GI = gastrointestinal.

A multitude of adjuvants for prolongation of peripheral nerve blocks have been investigated, but none have FDA approval for this purpose and because most are off-patent, they are unlikely to gain FDA approval in the absence of a vested-interest commercial sponsor. Moreover, few agents have been thoroughly investigated for potential neurotoxicity, and few published clinical trials have appropriate INDs or equivalent status. Based on our review, however, a few adjuvants that are widely utilized and broadly studied have been shown to be efficacious for prolongation of peripheral nerve blocks for postoperative analgesia with no clinical evidence of neurotoxicity. A summary of findings and recommendations is shown in Table 3, along with each agent’s criteria for inclusion.

Dexamethasone prolongs brachial plexus block with both intermediate- (168–343 minutes) and long-acting local anesthetics (730–1,306 minutes). There is conflicting information regarding dosing, given certain randomized controlled trials describe equivalence when utilizing high doses of systemic and perineural administration of dexamethasone; however, low doses of perineural dexamethasone (1–2mg) appear to prolong nerve block duration compared to equivalent or higher doses of IV dexamethasone (4mg). Further studies need to look at the efficacy of low perineural doses of dexamethasone to determine if less may be preferable to minimize toxicity and systemic effects. Buprenorphine (various doses) prolongs sciatic and brachial plexus blocks with long-acting local anesthetics by approximately 6–12 hours. Supra-clinical doses of dexamethasone and buprenorphine have demonstrated neurotoxicity in *in vitro* animal models; however, recent *in vivo* animal safety models show no adverse event levels [5] and potential neuroprotection and antihyperalgesic effects with clinically relevant dosing [111].

Both clonidine and dexmedetomidine likely prolong peripheral nerve blocks to a moderate degree. However, both clonidine and dexmedetomidine can cause bradycardia and hypotension in higher doses and should be used with caution. It should be noted that ongoing research suggests that each of these agents may demonstrate utility as part of multimodal perineural analgesia regimens [5, 7].

Magnesium has shown consistent efficacy for prolongation of peripheral nerve blocks, but there is little high-quality research supporting a clinically significant increased duration of analgesia. Animal models suggest that neuraxial administration of magnesium may be neurotoxic, and further *in vitro* and *in vivo* animal studies are necessary to clarify its safety profile prior to further perineural study or use in humans.

Head-to-head comparisons of local anesthetic peripheral nerve block adjuvants with promising safety and efficacy profiles will be useful in guiding future clinical study priorities and practice guidelines. In the absence of gold standards, studies of novel agents may best be conducted in comparison to both placebo and the agent established as “best in class”. In addition, many of the adjuvants reviewed here differ in their mechanisms of action and have the potential for additive or synergistic effects. Future research efforts should include further study of multimodal perineural analgesia, including the analgesic potential of agents administered in the absence of local anesthetics [2–6].

## Supporting Information

S1 File PRISMA checklist. All items corresponding to the checklist are displayed.
(DOC)

## References

[1] Ilfeld BM. Continuous peripheral nerve blocks: a review of the published evidence. Anesth Analg. 2011;113(4):904–25. 10.1213/ANE.0b013e3182285e01 21821511

[2] Williams BA, Hough KA, Tsui BY, Ibinson JW, Gold MS, Gebhart GF. Neurotoxicity of adjuvants used in perineural anesthesia and analgesia in comparison with ropivacaine. Reg Anesth Pain Med. 2011;36(3):225–30. 10.1097/AAP.0b013e3182176f70 21519308 PMC3085859

[3] Williams BA, Murinson BB, Grable BR, Orebaugh SL. Future considerations for pharmacologic adjuvants in single-injection peripheral nerve blocks for patients with diabetes mellitus. Reg Anesth Pain Med. 2009;34(5):445–57. 10.1097/AAP.0b013e3181ac9e42 19920420

[4] Williams BA, Schott NJ, Mangione MP, Ibinson JW. Perineural dexamethasone and multimodal perineural analgesia: how much is too much? Anesth Analg. 2014;118(5):912–4. 10.1213/ANE.0000000000000203 24781562

[5] Williams BA, Butt MT, Zeller JR, Coffee S, Pippi MA. Multimodal perineural analgesia with combined bupivacaine-clonidine-buprenorphine-dexamethasone: safe in vivo and chemically compatible in solution. Pain Med. 2015;16(1):186–98. 10.1111/pme.12592 25339320

[6] Williams BA, Ibinson JW, Mangione MP, Modrak RT, Tonarelli EJ, Rakesh H, et al Research priorities regarding multimodal peripheral nerve blocks for postoperative analgesia and anesthesia based on hospital quality data extracted from over 1,300 cases (2011–2014). Pain Med. 2015;16(1):7–12. 10.1111/pme.12609 25377071

[7] Williams BA, Ibinson JW, Mangione MP, Scanlan RL, Cohen PZ. Clinical benchmarks regarding multimodal peripheral nerve blocks for postoperative analgesia: observations regarding combined perineural midazolam-clonidine-buprenorphine-dexamethasone. Pain Med. 2015;16(1):1–6. 10.1111/pme.12599 25351887

[8] Neal JM, Brull R, Chan VW, Grant SA, Horn JL, Liu SS, et al The ASRA evidence-based medicine assessment of ultrasound-guided regional anesthesia and pain medicine: Executive summary. Reg Anesth Pain Med. 2010;35(2 Suppl):S1–9. 10.1097/AAP.0b013e3181d22fe0 20216019

[9] West S, King V, Carey TS, Lohr KN, McKoy N, Sutton SF, et al Systems to rate the strength of scientific evidence (Prepared by Research Triangle Institute-University of North Carolina Evidence-based Practice Center under contract No. 290-97-0011). Research Triangle Institute-University of North Carolina Evidence-based Practice C, United States. Agency for Healthcare R, Quality, editors. Rockville, MD: Agency for Healthcare Research and Quality; 2002.

[10] Oxford Centre for Evidence-based Medicine-Levels of Evidence (March 2009) [cited 2015 February 11]. Available from: http://www.cebm.net/oxford-centre-evidence-based-medicine-levels-evidence-march-2009/.

[11] Picard PR, Tramer MR, McQuay HJ, Moore RA. Analgesic efficacy of peripheral opioids (all except intra-articular): a qualitative systematic review of randomised controlled trials. Pain. 1997;72(3):309–18. 9313271 10.1016/s0304-3959(97)00040-7

[12] Leffler A, Frank G, Kistner K, Niedermirtl F, Koppert W, Reeh PW, et al Local anesthetic-like inhibition of voltage-gated Na(+) channels by the partial mu-opioid receptor agonist buprenorphine. Anesthesiology. 2012;116(6):1335–46. 10.1097/ALN.0b013e3182557917 22504149

[13] Koppert W, Ihmsen H, Korber N, Wehrfritz A, Sittl R, Schmelz M, et al Different profiles of buprenorphine-induced analgesia and antihyperalgesia in a human pain model. Pain. 2005;118(1–2):15–22. 16154698 10.1016/j.pain.2005.06.030

[14] Troster A, Ihmsen H, Singler B, Filitz J, Koppert W. Interaction of fentanyl and buprenorphine in an experimental model of pain and central sensitization in human volunteers. Clin J Pain. 2012;28(8):705–11. 10.1097/AJP.0b013e318241d948 22469638

[15] Bazin JE, Massoni C, Bruelle P, Fenies V, Groslier D, Schoeffler P. The addition of opioids to local anaesthetics in brachial plexus block: the comparative effects of morphine, buprenorphine and sufentanil. Anaesthesia. 1997;52(9):858–62. 9349066 10.1111/j.1365-2044.1997.174-az0311.x

[16] Candido KD, Franco CD, Khan MA, Winnie AP, Raja DS. Buprenorphine added to the local anesthetic for brachial plexus block to provide postoperative analgesia in outpatients. Reg Anesth Pain Med. 2001;26(4):352–6. 11464356 10.1053/rapm.2001.23931

[17] Candido KD, Winnie AP, Ghaleb AH, Fattouh MW, Franco CD. Buprenorphine added to the local anesthetic for axillary brachial plexus block prolongs postoperative analgesia. Reg Anesth Pain Med. 2002;27(2):162–7. 11915063 10.1053/rapm.2002.30671

[18] Candido KD, Hennes J, Gonzalez S, Mikat-Stevens M, Pinzur M, Vasic V, et al Buprenorphine enhances and prolongs the postoperative analgesic effect of bupivacaine in patients receiving infragluteal sciatic nerve block. Anesthesiology. 2010;113(6):1419–26. 10.1097/ALN.0b013e3181f90ce8 21042200

[19] Behr A, Freo U, Ori C, Westermann B, Alemanno F. Buprenorphine added to levobupivacaine enhances postoperative analgesia of middle interscalene brachial plexus block. J Anesth. 2012;26(5):746–51. 10.1007/s00540-012-1416-4 22644357

[20] Jadon A, Panigrahi MR, Parida SS, Chakraborty S, Agrawal PS, Panda A. Buprenorphine improves the efficacy of bupivacaine in nerve plexus block: A double blind randomized evaluation in subclavian perivascular brachial block. J Anaesthesiol Clin Pharmacol. 2009;25(2):207–10.

[21] Skledar SJ, Williams BA, Vallejo MC, Dalby PL, Waters JH, Glick R, et al Eliminating postoperative nausea and vomiting in outpatient surgery with multimodal strategies including low doses of nonsedating, off-patent antiemetics: is "zero tolerance" achievable? ScientificWorldJournal. 2007;7:959–77. 17619778 10.1100/tsw.2007.131 PMC5901347

[22] Williams BA, Kentor ML, Skledar SJ, Orebaugh SL, Vallejo MC. Routine multimodal antiemesis including low-dose perphenazine in an ambulatory surgery unit of a university hospital: a 10-year history. Supplement to: Eliminating postoperative nausea and vomiting in outpatient surgery with multimodal strategies including low doses of nonsedating, off-patent antiemetics: is "zero tolerance" achievable? ScientificWorldJournal. 2007;7:978–86. 17619779 10.1100/tsw.2007.132 PMC5901189

[23] Henao JP, Peperzak KA, Lichvar AB, Orebaugh SL, Skledar SJ, Pippi MA, et al Extrapyramidal symptoms following administration of oral perphenazine 4 or 8 mg: an 11-year retrospective analysis. Eur J Anaesthesiol. 2014;31(4):231–5. 10.1097/EJA.0000000000000048 24503705 PMC4365999

[24] Bourke DL, Furman WR. Improved postoperative analgesia with morphine added to axillary block solution. J Clin Anesth. 1993;5(2):114–7. 8476616 10.1016/0952-8180(93)90137-4

[25] Flory N, Van-Gessel E, Donald F, Hoffmeyer P, Gamulin Z. Does the addition of morphine to brachial plexus block improve analgesia after shoulder surgery? Br J Anaesth. 1995;75(1):23–6. 7669462 10.1093/bja/75.1.23

[26] Sternlo JE, Hagerdal M. Perineuronal morphine in intercostal block. Anaesthesia. 1992;47(7):613–5. 1626677 10.1111/j.1365-2044.1992.tb02338.x

[27] Racz H, Gunning K, Della Santa D, Forster A. Evaluation of the effect of perineuronal morphine on the quality of postoperative analgesia after axillary plexus block: a randomized double-blind study. Anesth Analg. 1991;72(6):769–72. 2035858 10.1213/00000539-199106000-00009

[28] Keskinbora K, Aydinli I. Perineural morphine in patients with chronic ischemic lower extremity pain: Efficacy and long-term results. J Anesth. 2009;23(1):11–8. 10.1007/s00540-008-0700-9 19234816

[29] Nishikawa K, Kanaya N, Nakayama M, Igarashi M, Tsunoda K, Namiki A. Fentanyl improves analgesia but prolongs the onset of axillary brachial plexus block by peripheral mechanism. Anesth Analg. 2000;91(2):384–7. 10910853 10.1097/00000539-200008000-00028

[30] Fanelli G, Casati A, Magistris L, Berti M, Albertin A, Scarioni M, et al Fentanyl does not improve the nerve block characteristics of axillary brachial plexus anaesthesia performed with ropivacaine. Acta Anaesthesiol Scand. 2001;45(5):590–4. 11309009 10.1034/j.1399-6576.2001.045005590.x

[31] Fletcher D, Kuhlman G, Samii K. Addition of fentanyl to 1.5% lidocaine does not increase the success of axillary plexus block. Reg Anesth. 1994;19(3):183–8. 7999653

[32] Kardash K, Schools A, Concepcion M. Effects of brachial plexus fentanyl on supraclavicular block. A randomized, double-blind study. Reg Anesth. 1995;20(4):311–5. 7577779

[33] Magistris L, Casati A, Albertin A, Deni F, Danelli G, Borghi B, et al Combined sciatic-femoral nerve block with 0.75% ropivacaine: effects of adding a systemically inactive dose of fentanyl. Eur J Anaesthesiol. 2000;17(6):348–53. 10928433 10.1046/j.1365-2346.2000.00685.x

[34] Moharari R, Sadeghi J, Khajavi M, Davari M, Mojtahedzadeh M. Fentanyl supplement expedites the onset time of sensory and motor blocking in interscalene lidocaine anesthesia. Daru. 2010;18(4):298–302. 22615632 PMC3304350

[35] Karakaya D, Buyukgoz F, Baris S, Guldogus F, Tur A. Addition of fentanyl to bupivacaine prolongs anesthesia and analgesia in axillary brachial plexus block. Reg Anesth Pain Med. 2001;26(5):434–8. 11561263 10.1053/rapm.2001.24675

[36] Sert H, Muslu B, Usta B, Colak N, Irem Demircioglu R, Gozdemir M. A comparison of articaine and fentanyl-supplemented articaine for hemodialysis fistula creation under ultrasound-guided axillary block. Ren Fail. 2011;33(3):280–4. 10.3109/0886022X.2011.560502 21401351

[37] Bhuvaneswari V, Wig J, Mathew PJ, Singh G. Post-operative pain and analgesic requirements after paravertebral block for mastectomy: A randomized controlled trial of different concentrations of bupivacaine and fentanyl. Indian J Anaesth. 2012;56(1):34–9. 10.4103/0019-5049.93341 22529417 PMC3327067

[38] Sindjelic RP, Vlajkovic GP, Davidovic LB, Markovic DZ, Markovic MD. The addition of fentanyl to local anesthetics affects the quality and duration of cervical plexus block: a randomized, controlled trial. Anesth Analg. 2010;111(1):234–7. 10.1213/ANE.0b013e3181e1e9ab 20519423

[39] Sabbe MB, Grafe MR, Mjanger E, Tiseo PJ, Hill HF, Yaksh TL. Spinal delivery of sufentanil, alfentanil, and morphine in dogs. Physiologic and toxicologic investigations. Anesthesiology. 1994;81(4):899–920. 7943841 10.1097/00000542-199410000-00017

[40] Werdehausen R, Braun S, Hermanns H, Kremer D, Kury P, Hollmann MW, et al The influence of adjuvants used in regional anesthesia on lidocaine-induced neurotoxicity in vitro. Reg Anesth Pain Med. 2011;36(5):436–43. 10.1097/AAP.0b013e318226ba62 21857277

[41] Braun H. Ueber die bedeutung des ephinephrine fur die chirurgie. Munch Med Wschr. 1903;50:352–3.

[42] Dogru K, Duygulu F, Yildiz K, Kotanoglu MS, Madenoglu H, Boyaci A. Hemodynamic and blockade effects of high/low epinephrine doses during axillary brachial plexus blockade with lidocaine 1.5%: A randomized, double-blinded study. Reg Anesth Pain Med. 2003;28(5):401–5. 14556129 10.1016/s1098-7339(03)00225-6

[43] Song JH, Shim HY, Lee TJ, Jung JK, Cha YD, Lee DI, et al Comparison of dexmedetomidine and epinephrine as an adjuvant to 1% mepivacaine in brachial plexus block. Korean J Anesthesiol. 2014;66(4):283–9. 10.4097/kjae.2014.66.4.283 24851163 PMC4028555

[44] Weber A, Fournier R, Van Gessel E, Riand N, Gamulin Z. Epinephrine does not prolong the analgesia of 20 mL ropivacaine 0.5% or 0.2% in a femoral three-in-one block. Anesth Analg. 2001;93(5):1327–31. 11682424 10.1097/00000539-200111000-00060

[45] Eledjam JJ, Deschodt J, Viel EJ, Lubrano JF, Charavel P, d'Athis F, et al Brachial plexus block with bupivacaine: effects of added alpha-adrenergic agonists: comparison between clonidine and epinephrine. Can J Anaesth. 1991;38(7):870–5. 1742820 10.1007/BF03036962

[46] Myers RR, Heckman HM. Effects of local anesthesia on nerve blood flow: studies using lidocaine with and without epinephrine. Anesthesiology. 1989;71(5):757–62. 2817471 10.1097/00000542-198911000-00021

[47] Kroin JS, Buvanendran A, Williams DK, Wagenaar B, Moric M, Tuman KJ, et al Local anesthetic sciatic nerve block and nerve fiber damage in diabetic rats. Reg Anesth Pain Med. 2010;35(4):343–50. 20607875 10.1097/aap.0b013e3181e82df0

[48] Neal JM. Effects of epinephrine in local anesthetics on the central and peripheral nervous systems: Neurotoxicity and neural blood flow. Reg Anesth Pain Med. 2003;28(2):124–34. 12677623 10.1053/rapm.2003.50024

[49] Kroin JS, Buvanendran A, Beck DR, Topic JE, Watts DE, Tuman KJ. Clonidine prolongation of lidocaine analgesia after sciatic nerve block in rats Is mediated via the hyperpolarization-activated cation current, not by alpha-adrenoreceptors. Anesthesiology. 2004;101(2):488–94. 15277933 10.1097/00000542-200408000-00031

[50] Tamsen A, Gordh T. Epidural clonidine produces analgesia. Lancet. 1984;2(8396):231–2. 10.1016/s0140-6736(84)90523-3 6146793

[51] Popping DM, Elia N, Marret E, Wenk M, Tramer MR. Clonidine as an adjuvant to local anesthetics for peripheral nerve and plexus blocks: a meta-analysis of randomized trials. Anesthesiology. 2009;111(2):406–15. 10.1097/ALN.0b013e3181aae897 19602964

[52] McCartney CJ, Duggan E, Apatu E. Should we add clonidine to local anesthetic for peripheral nerve blockade? A qualitative systematic review of the literature. Reg Anesth Pain Med. 2007;32(4):330–8. 17720118 10.1016/j.rapm.2007.02.010

[53] YaDeau JT, LaSala VR, Paroli L, Kahn RL, Jules-Elysee KM, Levine DS, et al Clonidine and analgesic duration after popliteal fossa nerve blockade: randomized, double-blind, placebo-controlled study. Anesth Analg. 2008;106(6):1916–20. 10.1213/ane.0b013e318172fe44 18499632

[54] Fournier R, Faust A, Chassot O, Gamulin Z. Perineural clonidine does not prolong levobupivacaine 0.5% after sciatic nerve block using the Labat approach in foot and ankle surgery. Reg Anesth Pain Med. 2012;37(5):521–4. 10.1097/AAP.0b013e3182606168 22854394

[55] Jaiswal R, Bansal T, Mehta S, Ahlawat G. A study to evaluate the effect of adding Clonidine to Ropivacaine for axillary plexus blockade. Asian J Pharm Clin Res. 2013;6(SUPPL.3):165–8.

[56] Kohli S, Kaur M, Sahoo S, Vajifdar H, Kohli P. Brachial plexus block: Comparison of two different doses of clonidine added to bupivacaine. J Anaesthesiol Clin Pharmacol. 2013;29(4):491–5. 10.4103/0970-9185.119147 24249986 PMC3819843

[57] Chakraborty S, Chakrabarti J, Mandal MC, Hazra A, Das S. Effect of clonidine as adjuvant in bupivacaine-induced supraclavicular brachial plexus block: A randomized controlled trial. Indian J Pharmacol. 2010;42(2):74–7. 10.4103/0253-7613.64498 20711369 PMC2907018

[58] Molnar RR, Davies MJ, Scott DA, Silbert BS, Mooney PH. Comparison of clonidine and epinephrine in lidocaine for cervical plexus block. Reg Anesth. 1997;22(2):137–42. 9089855 10.1016/s1098-7339(06)80032-5

[59] Trivedi V, Patel N. A comparative clinical study of injection clonidine versus midazolam in supraclavicular brachial plexus block for sedation and postoperative analgesia: a study of 60 cases. J Indian Med Assoc. 2010;108(9):563–7. 21510527

[60] Kamibayashi T, Maze M. Clinical uses of alpha2-adrenergic agonists. Anesthesiology. 2000;93(5):1345–9. 11046225 10.1097/00000542-200011000-00030

[61] Brummett CM, Hong EK, Janda AM, Amodeo FS, Lydic R. Perineural dexmedetomidine added to ropivacaine for sciatic nerve block in rats prolongs the duration of analgesia by blocking the hyperpolarization-activated cation current. Anesthesiology. 2011;115(4):836–43. 10.1097/ALN.0b013e318221fcc9 21666435 PMC3179561

[62] Memis D, Turan A, Karamanlioglu B, Pamukcu Z, Kurt I. Adding dexmedetomidine to lidocaine for intravenous regional anesthesia. Anesth Analg. 2004;98(3):835–40, table of contents. 14980948 10.1213/01.ane.0000100680.77978.66

[63] Abdallah FW, Brull R. Facilitatory effects of perineural dexmedetomidine on neuraxial and peripheral nerve block: a systematic review and meta-analysis. Br J Anaesth. 2013;110(6):915–25. 10.1093/bja/aet066 23587874

[64] Agarwal S, Aggarwal R, Gupta P. Dexmedetomidine prolongs the effect of bupivacaine in supraclavicular brachial plexus block. J Anaesthesiol Clin Pharmacol. 2014;30(1):36–40. 10.4103/0970-9185.125701 24574591 PMC3927290

[65] Fritsch G, Danninger T, Allerberger K, Tsodikov A, Felder TK, Kapeller M, et al Dexmedetomidine added to ropivacaine extends the duration of interscalene brachial plexus blocks for elective shoulder surgery when compared with ropivacaine alone: a single-center, prospective, triple-blind, randomized controlled trial. Reg Anesth Pain Med. 2014;39(1):37–47. 10.1097/AAP.0000000000000033 24317234

[66] Lin YN, Li Q, Yang RM, Mao ZX, Liu JC. Addition of dexmedetomidine to ropivacaine improves cervical plexus block. Acta Anaesthesiol Taiwan. 2013;51(2):63–6. 10.1016/j.aat.2013.06.001 23968656

[67] Marhofer D, Kettner SC, Marhofer P, Pils S, Weber M, Zeitlinger M. Dexmedetomidine as an adjuvant to ropivacaine prolongs peripheral nerve block: a volunteer study. Br J Anaesth. 2013;110(3):438–42. 10.1093/bja/aes400 23161360

[68] Rancourt MP, Albert NT, Cote M, Letourneau DR, Bernard PM. Posterior tibial nerve sensory blockade duration prolonged by adding dexmedetomidine to ropivacaine. Anesth Analg. 2012;115(4):958–62. 22826530 10.1213/ANE.0b013e318265bab7

[69] Swami SS, Keniya VM, Ladi SD, Rao R. Comparison of dexmedetomidine and clonidine (alpha2 agonist drugs) as an adjuvant to local anaesthesia in supraclavicular brachial plexus block: A randomised double-blind prospective study. Indian J Anaesth. 2012;56(3):243–9. 10.4103/0019-5049.98767 22923822 PMC3425283

[70] Zhang H, Zhou F, Li C, Kong M, Liu H, Zhang P, et al Molecular mechanisms underlying the analgesic property of intrathecal dexmedetomidine and its neurotoxicity evaluation: an in vivo and in vitro experimental study. PLoS One. 2013;8(2):e55556 10.1371/journal.pone.0055556 23409000 PMC3567091

[71] Tufek A, Kaya S, Tokgoz O, Firat U, Evliyaoglu O, Celik F, et al The protective effect of dexmedetomidine on bupivacaine-induced sciatic nerve inflammation is mediated by mast cells. Clin Invest Med. 2013;36(2):E95–102. 23544611 10.25011/cim.v36i2.19572

[72] Choi S, Rodseth R, McCartney CJ. Effects of dexamethasone as a local anaesthetic adjuvant for brachial plexus block: a systematic review and meta-analysis of randomized trials. Br J Anaesth. 2014;112(3):427–39. 10.1093/bja/aet417 24413428

[73] Biradar PA, Kaimar P, Gopalakrishna K. Effect of dexamethasone added to lidocaine in supraclavicular brachial plexus block: A prospective, randomised, double-blind study. Indian J Anaesth. 2013;57(2):180–4. 10.4103/0019-5049.111850 23825819 PMC3696267

[74] Ammar AS, Mahmoud KM. Effect of adding dexamethasone to bupivacaine on transversus abdominis plane block for abdominal hysterectomy: A prospective randomized controlled trial. Saudi J Anaesth. 2012;6(3):229–33. 10.4103/1658-354X.101213 23162395 PMC3498660

[75] Rasmussen SB, Saied NN, Bowens C Jr, Mercaldo ND, Schildcrout JS, Malchow RJ. Duration of upper and lower extremity peripheral nerve blockade is prolonged with dexamethasone when added to ropivacaine: a retrospective database analysis. Pain Med. 2013;14(8):1239–47. 10.1111/pme.12150 23755801

[76] Saritas A, Sabuncu C. Comparison of clinical effects of prilocaine, dexamethasone added to prilocaine and levobupivacaine on brachial plexus block. J Pak Med Assoc. 2014;64(4):433–6. 24864639

[77] Fredrickson MJ, Danesh-Clough TK, White R. Adjuvant dexamethasone for bupivacaine sciatic and ankle blocks: results from 2 randomized placebo-controlled trials. Reg Anesth Pain Med. 2013;38(4):300–7. 10.1097/AAP.0b013e318292c121 23698496

[78] Desmet M, Braems H, Reynvoet M, Plasschaert S, Van Cauwelaert J, Pottel H, et al I.V. and perineural dexamethasone are equivalent in increasing the analgesic duration of a single-shot interscalene block with ropivacaine for shoulder surgery: a prospective, randomized, placebo-controlled study. Br J Anaesth. 2013;111(3):445–52. 10.1093/bja/aet109 23587875

[79] Rahangdale R, Kendall MC, McCarthy RJ, Tureanu L, Doty R Jr, Weingart A, et al The effects of perineural versus intravenous dexamethasone on sciatic nerve blockade outcomes: a randomized, double-blind, placebo-controlled study. Anesth Analg. 2014;118(5):1113–9. 10.1213/ANE.0000000000000137 24686045

[80] Sondekoppam RV, Uppal V, Ganapathy S. Intravenous or perineural dexamethasone for interscalene brachial plexus block: the equivalence not yet proven. Br J Anaesth. 2014;112(1):175–6. 10.1093/bja/aet454 24318708

[81] Liu J, Richman KA, Grodofsky SR, Bhatt S, Huffman GR, Kelly JD 4th, et al Is there a dose response of dexamethasone as adjuvant for supraclavicular brachial plexus nerve block? A prospective randomized double-blinded clinical study. J Clin Anesth. 2015;27(3):237–42. 10.1016/j.jclinane.2014.12.004 25637938

[82] Ma R, Wang X, Lu C, Li C, Cheng Y, Ding G, et al Dexamethasone attenuated bupivacaine-induced neuron injury in vitro through a threonine-serine protein kinase B-dependent mechanism. Neuroscience. 2010;167(2):329–42. 10.1016/j.neuroscience.2009.12.049 20038443

[83] Dani C, Vestri V, Bertini G, Pratesi S, Rubaltelli FF. Toxicity of corticosteroids and catecholamines for mice neuronal cell cultures: Role of preservatives. J Matern Fetal Neonatal Med. 2007;20(4):325–33. 17437241 10.1080/14767050701227992

[84] Kim YH, Lee PB, Park J, Lim YJ, Kim YC, Lee SC, et al The neurological safety of epidural parecoxib in rats. Neurotoxicology. 2011;32(6):864–70. 10.1016/j.neuro.2011.05.011 21669221

[85] Canduz B, Aktug H, Mavioglu O, Erkin Y, Yilmaz O, Uyanikgil Y, et al Epidural lornoxicam administration—innocent. J Clin Neurosci. 2007;14(10):968–74. 17823047 10.1016/j.jocn.2006.10.006

[86] Guven M, Mert T, Gunay I. Effects of tramadol on nerve action potentials in rat: comparisons with benzocaine and lidocaine. Int J Neurosci. 2005;115(3):339–49. 15804720 10.1080/00207450590520948

[87] Sousa AM, Ashmawi HA, Costa LS, Posso IP, Slullitel A. Percutaneous sciatic nerve block with tramadol induces analgesia and motor blockade in two animal pain models. Braz J Med Biol Res. 2012;45(2):147–52. 22183244 10.1590/S0100-879X2011007500164 PMC3854253

[88] Bailard NS, Ortiz J, Flores RA. Additives to local anesthetics for peripheral nerve blocks: Evidence, limitations, and recommendations. Am J Health Syst Pharm. 2014;71(5):373–85. 10.2146/ajhp130336 24534592

[89] Alemanno F, Ghisi D, Fanelli A, Faliva A, Pergolotti B, Bizzarri F, et al Tramadol and 0.5% levobupivacaine for single-shot interscalene block: effects on postoperative analgesia in patients undergoing shoulder arthroplasty. Minerva Anestesiol. 2012;78(3):291–6. 21971437

[90] Kaabachi O, Ouezini R, Koubaa W, Ghrab B, Zargouni A, Ben Abdelaziz A. Tramadol as an adjuvant to lidocaine for axillary brachial plexus block. Anesth Analg. 2009;108(1):367–70. 10.1213/ane.0b013e31818e0c6b 19095875

[91] Sarsu S, Mizrak A, Karakurum G. Tramadol use for axillary brachial plexus blockade. J Surg Res. 2011;165(1):e23–7. 10.1016/j.jss.2010.09.032 21035132

[92] Mannion S, O'Callaghan S, Murphy DB, Shorten GD. Tramadol as adjunct to psoas compartment block with levobupivacaine 0.5%: a randomized double-blinded study. Br J Anaesth. 2005;94(3):352–6. 15608044 10.1093/bja/aei057

[93] Robaux S, Blunt C, Viel E, Cuvillon P, Nouguier P, Dautel G, et al Tramadol added to 1.5% mepivacaine for axillary brachial plexus block improves postoperative analgesia dose-dependently. Anesth Analg. 2004;98(4):1172–7, table of contents. 15041620 10.1213/01.ANE.0000108966.84797.72

[94] Kapral S, Gollmann G, Waltl B, Likar R, Sladen RN, Weinstabl C, et al Tramadol added to mepivacaine prolongs the duration of an axillary brachial plexus blockade. Anesth Analg. 1999;88(4):853–6. 10195537 10.1097/00000539-199904000-00032

[95] Kesimci E, Izdes S, Gozdemir M, Kanbak O. Tramadol does not prolong the effect of ropivacaine 7.5 mg/ml for axillary brachial plexus block. Acta Anaesthesiol Scand. 2007;51(6):736–41. 17425616 10.1111/j.1399-6576.2007.01308.x

[96] Omar AM, Mansour MA, Abdelwahab HH, Aboushanab OH. Role of ketamine and tramadol as adjuncts to bupivacaine 0.5% in paravertebral block for breast surgery: A randomized double-blind study. Egypt J Anaesth. 2011;27(2):101–5.

[97] Nishiyama T, Matsukawa T, Hanaoka K. Continuous epidural administration of midazolam and bupivacaine for postoperative analgesia. Acta Anaesthesiol Scand. 1999;43(5):568–72. 10342007 10.1034/j.1399-6576.1999.430514.x

[98] Malinovsky JM, Cozian A, Lepage JY, Mussini JM, Pinaud M, Souron R. Ketamine and midazolam neurotoxicity in the rabbit. Anesthesiology. 1991;75(1):91–7. 2064066 10.1097/00000542-199107000-00015

[99] Erdine S, Yucel A, Ozyalcin S, Ozyuvaci E, Talu GK, Ahiskali B, et al Neurotoxicity of midazolam in the rabbit. Pain. 1999;80(1–2):419–23. 10204757 10.1016/s0304-3959(98)00240-1

[100] Demirel E, Ugur HC, Dolgun H, Kahilogullari G, Sargon ME, Egemen N, et al The neurotoxic effects of intrathecal midazolam and neostigmine in rabbits. Anaesth Intensive Care. 2006;34(2):218–23. 16617644 10.1177/0310057X0603400204

[101] Vastani N, Seifert B, Spahn DR, Maurer K. Sensitivities of rat primary sensory afferent nerves to magnesium: implications for differential nerve blocks. Eur J Anaesthesiol. 2013;30(1):21–8. 10.1097/EJA.0b013e32835949ab 23138572

[102] Elshamaa HA, Ibrahim M, Eldesuky HL. Magnesium sulfate in femoral nerve block, does postoperative analgesia differ? A comparative study. Egypt J Anaesth. 2014;30(2):169–73.

[103] Lee AR, Yi HW, Chung IS, Ko JS, Ahn HJ, Gwak MS, et al Magnesium added to bupivacaine prolongs the duration of analgesia after interscalene nerve block. Can J Anaesth. 2012;59(1):21–7. 10.1007/s12630-011-9604-5 22012543

[104] Gunduz A, Bilir A, Gulec S. Magnesium added to prilocaine prolongs the duration of axillary plexus block. Reg Anesth Pain Med. 2006;31(3):233–6. 16701189 10.1016/j.rapm.2006.03.001

[105] Dogru K, Yildirim D, Ulgey A, Aksu R, Bicer C, Boyaci A. Adding magnesium to levobupivacaine for axillary brachial plexus block in arteriovenous fistule surgery. Bratisl Lek Listy. 2012;113(10):607–9. 23094900 10.4149/bll_2012_136

[106] Saeki H, Matsumoto M, Kaneko S, Tsuruta S, Cui YJ, Ohtake K, et al Is intrathecal magnesium sulfate safe and protective against ischemic spinal cord injury in rabbits? Anesth Analg. 2004;99(6):1805–12, table of contents. 15562076 10.1213/01.ANE.0000138039.04548.3D

[107] Albrecht E, Kirkham KR, Liu SS, Brull R. The analgesic efficacy and safety of neuraxial magnesium sulphate: a quantitative review. Anaesthesia. 2013;68(2):190–202. 10.1111/j.1365-2044.2012.07337.x 23121635

[108] Weber WV, Jawalekar KS, Jawalekar SR. The effect of ketamine on nerve conduction in isolated sciatic nerves of the toad. Neurosci Lett. 1975;1(2):115–20. 19604763 10.1016/0304-3940(75)90055-5

[109] Lee IO, Kim WK, Kong MH, Lee MK, Kim NS, Choi YS, et al No enhancement of sensory and motor blockade by ketamine added to ropivacaine interscalene brachial plexus blockade. Acta Anaesthesiol Scand. 2002;46(7):821–6. 12139537 10.1034/j.1399-6576.2002.460711.x

[110] Borgbjerg FM, Svensson BA, Frigast C, Gordh T Jr. Histopathology after repeated intrathecal injections of preservative-free ketamine in the rabbit: a light and electron microscopic examination. Anesth Analg. 1994;79(1):105–11. 8010418 10.1213/00000539-199407000-00020

[111] An K, Elkassabany NM, Liu J. Dexamethasone as adjuvant to bupivacaine prolongs the duration of thermal antinociception and prevents bupivacaine-induced rebound hyperalgesia via regional mechanism in a mouse sciatic nerve block model. PLoS One. 2015;10(4):e0123459 10.1371/journal.pone.0123459 25856078 PMC4391940

